# Supplementary material for: Development of a computer-aided design software for the quantitative evaluation of aesthetic damage
Source: PLoS One. 2019 Dec 18;14(12):e0226322. doi: 10.1371/journal.pone.0226322 (PMC6919621; doi:10.1371/journal.pone.0226322)
Supplement: S1 Code site — 1. (ZIP) [file pone.0226322.s001.zip › [EESC_jr][Projeto]Relatorio_07_21/reports/templates_zip/gentelella-master/vendors/DateJS/reports/lcov-report/index.html]

Code coverage report for All files


# Code coverage report for All files

## Statements: 82.29% (1385 / 1683)      Branches: 70.61% (793 / 1123)      Functions: 83.48% (288 / 345)      Lines: 82.5% (1377 / 1669)      Ignored: none

| File |  | Statements |  | Branches |  | Functions |  | Lines |  |
| --- | --- | --- | --- | --- | --- | --- | --- | --- | --- |
| core/ |  | 82.29% | (1385 / 1683) | 70.61% | (793 / 1123) | 83.48% | (288 / 345) | 82.5% | (1377 / 1669) |

Generated by istanbul at Sun Nov 16 2014 12:08:33 GMT-0800 (PST)
